# Intraosseous hair-induced cyst-like lesion of the maxilla associated with trichotillomania: first reported case and review of the literature

**DOI:** 10.1007/s10006-025-01495-4

**Published:** 2026-01-07

**Authors:** Jay Saepoo, Steven Dorris, Kittiphoj Tikkhanarak, Sherry Timmons, Nidhi Handoo, John Hellstein, Adam Holton, Emily Lanzel

**Affiliations:** 1 Department of Oral Pathology, Radiology and Medicine, College of Dentistry, The University of Iowa, 801 Newton Rd., Iowa city, IA USA; 2 Holton Oral and Maxillofacial Surgery, 820 Grand Ave, Spencer, IA 51301 USA

**Keywords:** Trichotillomania, Cyst, Foreign body reaction, Hair, Oral hair

## Abstract

Heterotopic hair in the oral cavity is a rare condition, with possible etiologies including prior skin graft reconstruction, oral and maxillofacial trauma, or, in some clinical context, no identifiable cause. We report a case of a patient with trichotillomania who presented with a persistent sinus track opening between teeth #9 and #10 and a well-defined periradicular lesion in the area. Microscopically, it revealed a partially lined cyst-like entity containing terminal hairs, associated with a foreign body-type giant cell reaction. The patient admitted to a habit of repeatedly pulling and plucking her hair, placing it in her mouth, and occasionally pressing it against the area of concern. To our knowledge, this is the first reported case of an intraosseous hair-induced cyst-like entity with foreign body reaction in the anterior maxilla associated with trichotillomania.

## Introduction

In DSM − 5 (Diagnostic and Statistical Manual of Mental Disorders), under Obsessive-Compulsive and Related Disorders category, trichotillomania is a psychological disorder characterized by repetitive hair pulling, with an estimated adult prevalence of approximately 1.7%, affecting men and women equally. This condition is often associated with significant distress and comorbid mental health disorders, including anxiety, depression, obsessive-compulsive disorder, post-traumatic stress disorder, and attention-deficit/hyperactivity disorder [1, 2].

Heterotopic hair within the oral cavity is a rare phenomenon. Previous reports have documented heterotopic hair growth in the facial gingiva, palatal gingiva, buccal mucosa, and dorsal tongue [3–7], or in the areas of prior skin graft reconstruction [8]. In most of these cases, no clear etiology for intraoral hair growth was identified [3, 4, 7].

Here, we report a case of an intraosseous hair-induced cyst with foreign body reaction in a patient with trichotillomania. To our knowledge, this is the first reported case of such a lesion in the anterior maxilla associated with trichotillomania.

## Case report

A 64-year-old female presented to a private oral and maxillofacial surgery clinic for evaluation of a persistent sinus track opening on the facial aspect between teeth #9 and #10. Clinical examination revealed soft tissues within normal limits, with no tenderness on palpation. There was no evidence of purulent, serous or serosanguinous discharge. Percussion testing elicited no pain, and vitality testing of the involved teeth showed normal responses. There was no evidence of cleft lip or cleft palate, and the patient denied any history of previous palatal surgery.

**Fig. 1:**
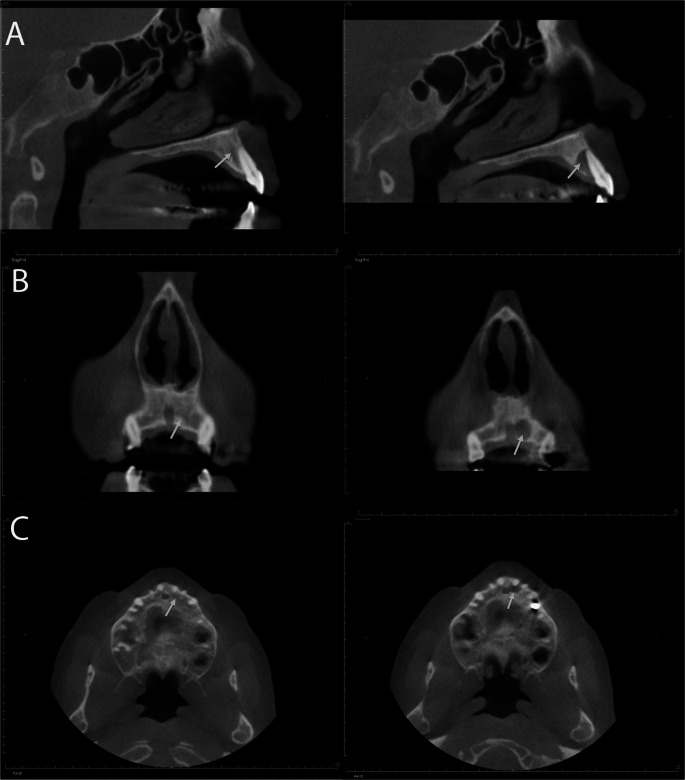
Radiographic findings of an intraosseous hair-induced cyst-like lesion between teeth #9 and #10. The region of interest from November 2023 (left-sided) and February 2025 (right-sided) demonstrated no evidence of pathosis found in November 2023 slices compared to the appearance of hydraulic-appearing, hypodense entity in February 2025. (Panel **A**) Sagittal view, (Panel **B**) Coronal view, and (Panel **C**) Axial view

The area of the chief complaint was further evaluated using cone beam computed tomography (CBCT) of the maxilla. Radiographic examination revealed a well-defined, hydraulic-appearing hypodense entity. The entity was interproximal and palatal to the maxillary left incisors with discontinuities in the adjacent facial and palatal cortices. Portions of the entity appeared just distal to the nasopalatine canal such that visualization of the cortication of the canal was lost in this region. The surrounding bone appeared mildly sclerotic. A previous CBCT from 15 months prior showed no evidence of the entity. (Fig. 1)

An excisional biopsy was performed under local anesthesia using 2% lidocaine with epinephrine (1:100,000). Intraoperatively, the area of concern revealed an intrabony cyst-like cavity with hair-like structures. The biopsy specimen was submitted for histopathological examination at the Surgical Oral Pathology Laboratory, College of Dentistry, University of Iowa. Given the vitality of the adjacent teeth on clinical examination, radiographic findings, and intraoperative findings, the differential diagnoses provided by clinician included an intraosseous epidermal inclusion cyst or a dermoid cyst.

**Fig. 2:**
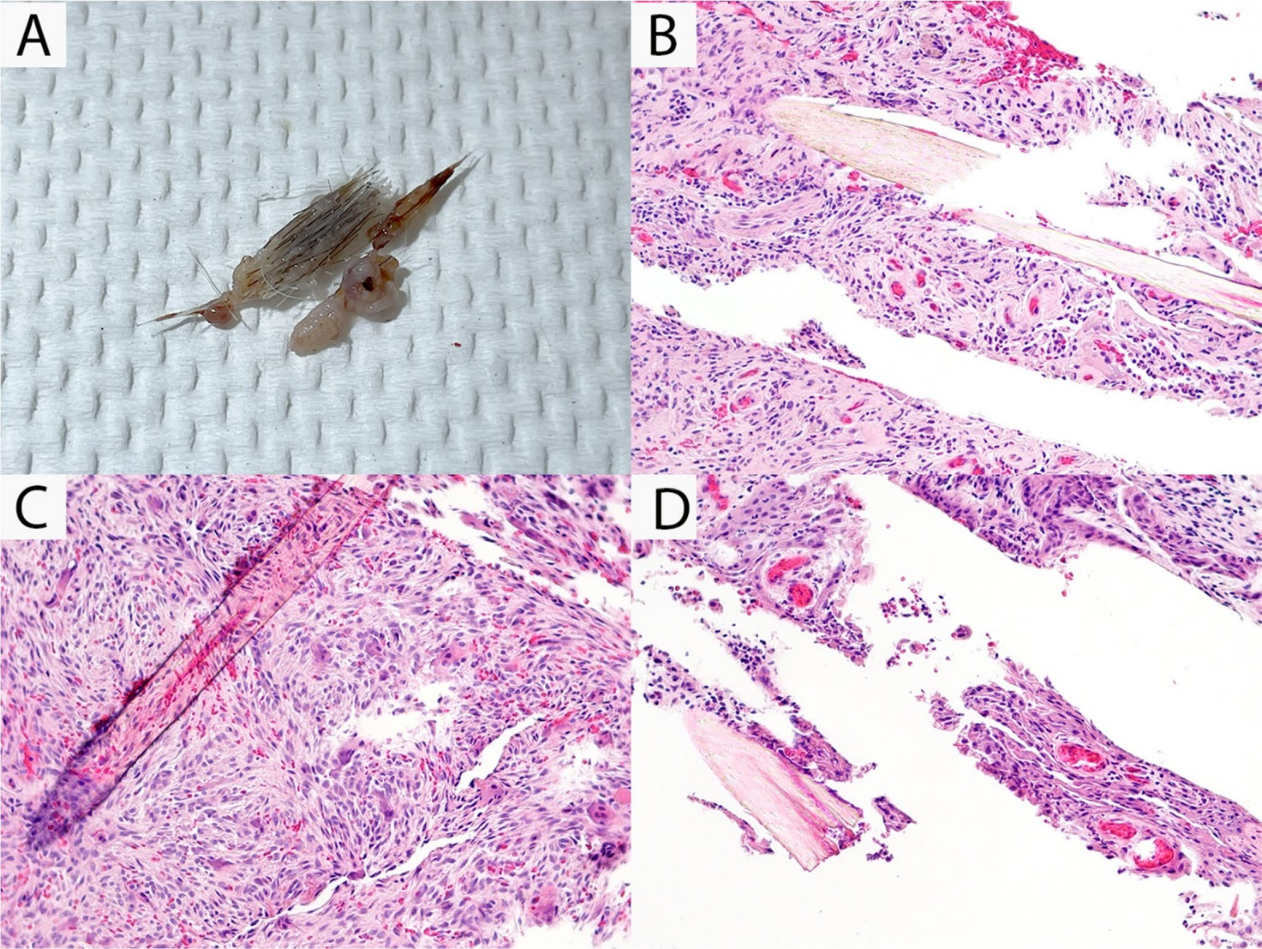
Gross and histopathological examination from an excisional biopsy of an intraosseous hair-induced cyst-like lesion between teeth #9 and #10. (**A**) Gross examination revealed fragments of irregularly shaped soft tissues with hair structures, (**B**) Microscopic examination demonstrated reactive variably dense fibrocollagenous connective tissue containing terminal hairs associated with acute and chronic inflammation and small vessels and extravasated erythrocytes, (**C**) Terminal hair was associated with foreign-body multinucleated giant cell reaction, and (**D**) Fragments of non-keratinizing squamous epithelial lining were observed associated with hairs

Gross examination of the biopsy specimen revealed irregularly shaped soft tissue fragments containing hair shafts with follicles (Fig. 2A). Microscopic examination demonstrated fragments of thin, non-keratinizing stratified squamous epithelium associated with numerous terminal hairs. Additionally, there was focal proliferation of plump, spindle-shaped fibroblasts within a richly vascular stroma, along with numerous large multinucleated giant cells (Fig. 2B-D). The initial final diagnosis was “a hair-producing cyst of undetermined origin”.

## Discussion

This case highlights unusual histopathological findings in a well-defined hypodense interproximal and palatal lesion of the anterior maxillary teeth, initially diagnosed as “a hair-producing cyst of an undetermined origin”. In typical clinical scenarios, the differential diagnoses for well-defined periradicular or interradicular lesions include endodontic and non-endodontic pathoses. Among non-endodontic lesions, the most common entities are odontogenic keratocyst and nasopalatine duct cyst [9]. In the present case, the hypodense lesion involved the interproximal region of the anterior maxillary teeth, extended to the palatal aspect, and demonstrated a possibility of connection and loss of nasopalatine canal cortication (Fig. 1). Based on these radiographic and clinical findings, odontogenic keratocyst (OKC), nasopalatine duct cyst (NPDC), lateral periodontal cyst (LPC), and other benign odontogenic tumors, such as ameloblastoma and central odontogenic fibroma, were considered. However, given to the intraoperative findings during excisional biopsy, it revealed hair structures within the cyst-like cavity—an uncommon feature in such lesions. This unexpected observation prompted consideration of rare diagnoses, including intraosseous epidermal inclusion cyst [10, 11] and dermoid cyst [12]. Although no prior reports of intraosseous teratoma of the jaw bones exist, teratomas involving other bones have been documented and could also be considered as a differential diagnosis in this case [13, 14].

Histologically, the specimen consisted of fragments of variably dense fibrocollagenous connective tissue with prominent acute and chronic inflammatory infiltrates. Within the reactive connective tissue, terminal hair shafts associated with a foreign body–type multinucleated giant cell reaction were identified. Focal fragments of non-keratinizing squamous epithelium were also observed (Fig. 2). The histopathological features of OKC, NPDC, LPC, and other odontogenic tumors in the original differential diagnoses were not present. Although terminal hair and fragments of epithelial lining were observed, other features of cysts/tumors known to produce hairs were also not seen. Instead, the findings were most consistent with a foreign body reaction to hair, characterized by dense acute and chronic inflammation with multinucleated giant cell response. On this basis, an initial diagnosis of a “hair-producing cyst of undetermined origin” was rendered. Given the unusual and previously unreported nature of this entity, the oral pathologists requested that the clinician re-evaluate the patient’s history and re-examined to investigate potential sources of hair within the lesion, in order to achieve an appropriate clinico-pathological correlation.

It is extremely rare and unusual to observe heterotopic hair growth within oral cavity. As reported in most previous studies, the etiology of such occurrences remains unclear. Some authors have proposed that this phenomenon may result from aberrant embryonic fusion of ectodermal tissues or developmental defects [3–7]. Table 1 summarizes the reported cases of heterotopic hair growth in the oral cavity. As shown in the table, one report described hair overgrowth arising from a previous skin graft at a surgical site. Therefore, it is critical to rule out any prior history of palatal or oral surgery, including skin grafting procedures, that might account for intraoral hair growth [8], as well as history of orofacial trauma that could have introduced hair follicles or skin appendages into the oral cavity.

**Table 1:** Summary of reported intraoral heterotopic hair growth

Author(s), Year of Publication (Reference)	Demographics		Location(s)	Possible Etiology	Treatment	Additional Information
	Age at diagnosis	Sex				
Miles AE, 1960 [5]	57	Male	Buccal mucosa, at the level of the parotid papilla (posterior), 0.5 cm in size	Unknown etiology	Not applicable	The author proposed that heterotopic hair may result from aberrant embryonic fusion of the skin ectoderm.
Baughman RA et al., 1980 [4]	45	Male	Attached gingiva of teeth #26–27, 3.5 cm in size	Unknown etiology: the patient has a habit of “plucked hair”	Excision	The patient noticed hair growth in the area since teenager. He usually removed the hair with tweezers, but it would regrow.
Fetkowska-Mielnik KF et al., 1986 [7]	13	Male	Multiple sites: buccal and palatal aspects of the upper right quadrant teeth, 0.4–0.6 cm in size	Unknown etiology	Hair removal with surrounding oral mucosa.	- The patient reported hair growth one year ago, which recurred two months after removal. - The patient had underlying alopecia areata; the authors noted that as treatment progressed, oral hair regrowth decreased. - The authors proposed “a developmental defect” as the possible underlying cause
Agha-Hosseini F et al., 2007 [3]	11	Male	Multiple sites: buccal aspects of upper and lower incisors, interdental papillae of lower incisors, dorsal surface of the tongue, and oropharynx, 0.8–0.9 cm in size	Unknown etiology	Incisional biopsy with modified Widman flap	The authors stated that there was no attachment to the underlying structures, and the lesion appeared to originate from the free gingival margin.
Rochefort J, et al., 2016 [6]	30	Male	Middle dorsal of tongue	Unknown etiology	Excision	The patient reported rapid hair regrowth at the site a few days after self-removal. Consequently, he requested surgical excision.
Bains P and Mahajan A, 2017 [8]	40	Male	Palate, 2 cm x 3 cm in size.	Palatal reconstruction with thigh flap (skin graft)	Laser therapy was offered; however, the patient declined the treatment.	None
Femiano F et al., 2009 [17] and Zhurakivska K et al., 2020 [18]	Same patient: first noticed at age 19, with recurrence at age 25.	Female	First presentation (Age 19): palatal aspect of the gingival sulcus of tooth #8, 0.3–0.4 cm in size. Second presentation (Age 25): sulcus of teeth #8 and #9; at one-year follow-up, numerous hairs were noted at teeth #4, #5, #12, #13, #14, and #26.	High circulating testosterone in polycystic ovary syndrome, with a persistent structural defect, contributed to recurrence after six years.	- Excision for the first presentation. - Remove hairs with incisional biopsy of the involved gingival sulcus	The authors stated that recurrence of intraoral hair growth after the first presentation was possibly caused by persistent structural defects of the ectoderm, which contributed to hair production.
The present study, 2025	64	Female	Intraosseous lesion at the palatal aspect of periapical region of teeth #9 and #10	Relapsed trichotillomania: the patient pulled her hair and had a habit of placing and chewing it in her mouth (palatal side), accompanied by deep bite occlusion.	Excision biopsy of the intraosseous lesion.	The patient had controlled trichotillomania for several years but experienced a relapse following the loss of a household member, which affected her behavioral and psychosocial well-being.

However, in the present case, the patient denied any history of trauma, prior surgery, or procedures that could account for these findings. Interestingly, she instead reported a history of trichotillomania, characterized by habitual hair pulling and plucking. She admitted to occasionally placing the pulled hairs in her mouth, chewing or nibbling on them, and pressing them into the palatal site. She also exhibited a relatively deep bite, which may have facilitated this habit, leading to trapping of hairs in the palatal region, the subsequent development of a periapical lesion involving teeth #9 and #10, and eventually the formation of a sinus track opening on the facial aspect, corresponding to her chief complaint. She further stated that although she had controlled her trichotillomania for many years, the habit has returned about 18 months previously following the loss of a household member. This behavioral pattern provides a plausible explanation for how hair became entrapped at the site over time.

Given these clinical and behavioral findings, the most likely pathogenesis involves repeated mechanical irritation and microtrauma to the oral mucosa, allowing hair fragments to penetrate and become embedded in the soft tissue. These foreign materials subsequently triggered localized, persistent acute and chronic inflammatory responses. Over time, the inflammation may have contributed to focal osteolysis of the underlying bone, creating a pathway for hair to migrate into the intraosseous compartment. Once within the bone, the retained hair acted as a persistent foreign body, perpetuating inflammation. As a consequence, a foreign body reaction developed, characterized by localized acute and chronic inflammation and fibrous encapsulation around the hair, which ultimately resulted in the formation of a cystic lesion [15, 16].

To our knowledge, this is the first reported case of intraosseous hair entrapment resulting in a hair-induced cyst-like entity as a localized foreign body reaction. Previous reports have primarily described soft tissue involvement, either following surgical reconstruction with skin grafts [8], the possible association with alopecia areata [7], high circulating testosterone in polycystic ovary syndrome [17], or as lesions of undetermined origin in the buccal mucosa, tongue, or gingiva [3–7, 18]. In conclusion, we report a rare case of an intraosseous hair-induced cyst-like entity in the anterior maxilla. This case also emphasizes the significance of anamnesis in determining possible etiology of rare entities. In this case, trichotillomania was identified as a potential contributing factor to the development of the lesion.

## Data Availability

No datasets were generated or analyzed during the current study.
